# Pre-specified Anxiety Predicts Future Decision-Making Performances Under Different Temporally Constrained Conditions

**DOI:** 10.3389/fpsyg.2019.01544

**Published:** 2019-07-09

**Authors:** Takahiro Soshi, Mitsue Nagamine, Emiko Fukuda, Ai Takeuchi

**Affiliations:** ^1^Graduate School of Frontier Biosciences, Osaka University, Osaka, Japan; ^2^Institute for Liberal Arts, Tokyo Institute of Technology, Tokyo, Japan; ^3^Department of Industrial Engineering and Economics, School of Engineering, Tokyo Institute of Technology, Tokyo, Japan; ^4^College of Economics, Ritsumeikan University, Kyoto, Japan

**Keywords:** decision-making, Iowa Gambling Task, anxiety, temporal pressure, prospective approach

## Abstract

In real-life circumstances, people occasionally require making forced decisions when encountering unpredictable events and situations that yield socially and privately unfavorable consequences. In order to prevent future negative consequences, it is beneficial to successfully predict future decision-making behaviors based on various types of information, including behavioral traits and/or psychological states. For this prospective purpose, the present study used the Iowa Gambling Task, which simulates multiple aspects of real-life decision-making processes, such as choice preference, selection and evaluation of output feedback, and investigated how anxiety profiles predict decision-making performances under conditions with different temporal pressures on task execution. To conduct a temporally causal analysis, we assessed the trait and state anxiety profiles of 33 young participants prior to the task and analyzed their subsequent decision-making performances. We separated two disadvantageous card decks with high rewards and losses into high- and middle-risk decks, and calculated local performance indexes for decision-making immediately after salient penalty events for the high-risk deck in addition to traditional global performance indexes concerning overall trial outcomes such as final winnings and net scores. For global decision-making, higher trait anxiety predicted more risky choices solely in the self-paced condition without temporal pressure. For local decision-making, state anxiety predicted risk-taking performances differently in the self- and forced-paced conditions. In the self-paced condition, higher state anxiety predicted higher risk-avoidance. In the forced-paced condition, higher state anxiety predicted more frequent choices of the middle-risk deck. These findings suggest not only that pre-specified anxiety profiles can effectively predict future decision-making behaviors under different temporal pressures, but also newly indicate that behavioral mechanisms for moderate risk-taking under an emergent condition should be focused on to effectively prevent future unfavorable consequences when actually encountering negative events.

## Introduction

People often face various unpredictable events and must decide acts in daily-life situations. In particular, when events are emotionally salient under situational pressure and uncertainty, we may often have difficulty in stably making decisions to obtain positive consequences. To lead a smooth social life, it is advantageous to be able to predict future decision-making performances based on individual behavioral and psychological profiles before actually encountering negative events. Along with the somatic marker hypothesis, which posits that physiological signals anticipatorily affect decision-making under information uncertainty (Damasio, 1996; Bechara et al., 2003; Werner et al., 2013), we hypothesize that decision-making is an adaptive product of the interaction between situational and individual psychological factors, and examine how individual psychological profiles predict future decision-making behaviors.

To examine human decision-making processes in real-life situations in an experimental context, we used the Iowa Gambling Task (IGT), which has been frequently utilized during the past 20 years (Bechara et al., 1994; Chiu et al., 2018). The decision-making process instantiated in the IGT is defined as the executive function that among current lists of choice options that are perceived and/or stored in a short-term memory, people voluntarily select the best option (Bechara et al., 1998). The main characteristics of the IGT consist of the following three factors: (1) probabilistic emotional events of reward and loss, (2) information ambiguity (e.g., ratios between reward and loss) in task execution, and (3) reinforcement learning of an anticipatory decision-making strategy. In each trial of the IGT, participants are required to select one card from advantageous or disadvantageous card decks to maximize the sum of rewards in all trials. Participants are not initially provided with information about the compositions of rewards and losses in each deck nor its probabilities and must learn and anticipate deck types by feedback information about current rewards and penalties. Developing or modifying a decision-making strategy is iteratively based on three sub-processes (Paulus, 2005). The first is assessment of preference for the options, in which an individual assigns advantageous and/or disadvantageous values to behavioral options in an anticipatory manner. The second is execution of selectional action, where individuals must actually select one of the options and inhibit the other options. The third is evaluation of decision-making outcomes by comparing realized outcomes with their anticipation in order to reinforce or modify their preferences and choice patterns. Decision-making in the IGT tends to be distinguished from other executive functions such as cognitive switching and inhibition (Bechara et al., 1998; Bechara, 2004; Toplak et al., 2010); however, there is still controversy regarding whether there is a clear distinction between them (Fellows, 2007; Ouerchefani et al., 2017). As argued by Ernst et al. (2002), because the IGT includes both decision-making *per se*, such as execution of selection (Paulus, 2005), and anticipation of reward and loss as an emotional feedback, it activates not only the dorsolateral prefrontal and anterior cingulate cortices for executive functions such as attention (Ernst et al., 2002) but also the ventromedial prefrontal cortex and amygdala for emotional regulation (Bechara et al., 1998, 2003; Bechara, 2004).

One of the factors affecting decision-making includes temporal parameters (Bowman et al., 2005; Cella et al., 2007; DeDonno and Demaree, 2008; Madan et al., 2015). In real-life circumstances, we often face situations requiring decision-making not only with comfort but also under time constraints of short duration. Because temporal pressure allows limited resources for online psychological processing, it may affect wide aspects of decision-making such as option assessment, action execution, and outcome evaluation (Paulus, 2005). Cella et al. (2007) examined how external temporal pressure affected decision-making processes in the IGT. They divided participants into three groups and externally imposed 2- and 4-s temporal constraints on deck selection in two groups. Compared to the control group without temporal constraints, the participants with the 2-s constraint more frequently selected disadvantageous decks even as trial blocks advanced.

Internal temporal pressure also affects decision-making. DeDonno and Demaree (2008) did not expose participants to external temporal pressure; however, they manipulated the perceived time pressure for the task by presenting the explicit message that the time available for deck selection was not sufficient for successful task execution. Compared to the control group, the pressured group more frequently selected disadvantageous decks. Studies with clinical populations have also shown supportive evidence for the relationship between internal temporal pressure and decision-making. People with obsessive-compulsive disorder (OCD) show behavioral impulsivity (Nielen et al., 2002; Chamberlain et al., 2006; Benatti et al., 2014; Grassi et al., 2015), which is defined as the trait of spontaneously making temporally pressured or rapid responses without considering unfavorable consequences (Moeller et al., 2001). Patients with OCD tend to make risky choices (Cavedini et al., 2002; Starcke et al., 2010; Grassi et al., 2015; but for an opposing perspective, see Glicksohn et al., 2007) because of abnormal functional connections between the basal ganglia and ventromedial prefrontal cortex in the emotional regulation pathway (Rapoport, 1990). Grassi et al. (2015), for example, recruited participants diagnosed with OCD and compared their decision-making in the IGT with that of the control participants. Those with OCD had higher impulsivity scores (in particular, attentional, and non-planning impulsivity) than did the control participants, and they did not modify disadvantageous selections even during later trial blocks, as observed in a previous study (Starcke et al., 2010). Taken together, external and internal temporal pressures change decision-making in diverse populations.

Another influential factor is anxiety, which is evoked by subjective uncertainty to future-oriented negative situations and events (Cannistraro and Rauch, 2003; Mueller et al., 2010) and is occasionally experienced under threat; it is accompanied by autonomic physiological reactions and/or cognitive negativity bias (Robinson et al., 2013; Wiedemann, 2015). Future-oriented uncertainty, in particular, is an important environmental aspect that evokes anxiety and is a fundamental dimension of decision-making processes because of iteratively promoting reinforcement learning for solving uncertainty in an anticipatory manner (Paulus, 2005). The close relationship between anxiety and decision-making is also supported by neuroimaging findings that the ventromedial prefrontal cortex and amygdala are related to anxiety in an overlapping manner with decision-making (Tian et al., 2016; Brinkmann et al., 2018).

Anxiety is generally sub-divided into trait and state profiles (Spielberger et al., 1970; Spielberger, 1989). Trait anxiety is a stable personality profile characterized by a disposition to easily experiencing anxiety states. State anxiety is a relatively short-term emotional state under stress, consisting of transient feelings of tension and apprehension and an elevated automatic nervous response. Trait and state anxiety adversely or advantageously affect decision-making (Robinson et al., 2013). Starcke et al. (2008) separated participants into two groups with and without a prospective stressful speech task that was scheduled before a decision-making task, but was not actually conducted. The stress group, compared to the control group, reported higher state anxiety scores and attenuated risk-avoidance. That is, long-term stress related to future tasks may trigger intrusive thoughts and reduce memory resources for decision-making, resulting in less risk-avoidance (Ashcraft and Kirk, 2001).

On the other hand, anxiety can reversely promote conservative risk-avoidance (Mather et al., 2009; Clark et al., 2012). Clark et al. (2012) exposed participants to an electrical shock, a threat stimulus (Kuriyama et al., 2011), during each trial of a decision-making task and compared the performances with those in the safe condition without shocks. Participants were more risk-avoidant in the stress condition than in the safe condition, suggesting that aversive shocks were not intrusive and instead enhanced the interoception of physical responses or somatic markers for negative consequences or increased sensitivity to future threats by the automatic activation of emotional neural correlates such as the amygdala (Jiang et al., 2009) to promote risk-avoidance. Such risk-avoidance has also been persistently observed in a specific clinical population. Mueller et al. (2010) recruited individuals with generalized anxiety disorder (GAD), which is characterized by intense future-oriented anxious traits, and compared their decision-making behaviors in the IGT with those of controls. Those with GAD successively increased risk-avoidance performances compared to the controls as the trials advanced. These findings suggest that trait and state anxiety affect decision-making in divergent ways.

The effects of temporal pressure and anxiety may not be independent during decision-making in the IGT. Anxiety tends to be related to speeded-up mental processing such as racing thoughts, where thinking is accelerated subjectively (Pronin and Jacobs, 2008; Keizer et al., 2014; Aho, 2018). Thus, when people with different anxiety traits are exposed to externally temporally pressured conditions, they may respond differently to temporal pressure, yielding different patterns of decision-making processes. As has been initially argued in this chapter, from the prospective standpoint of preventing future negative consequences, it is beneficial to predict individual future decision-making behaviors before actually encountering salient negative events under emergent conditions. Such predictive benefits are more crucial for potential victims in social circumstances, when negative events are socially problematic, such as financial and billing frauds (Deevy et al., 2012).

Trait and state anxiety may predict different aspects of decision-making processes (Bishop, 2007; Tian et al., 2016). The IGT may present a conflicting situation between long- and short-term anxiety because of the alternation of reward and loss in an ambiguous manner. By continuously selecting low-risk decks with low reward and loss, people can effectively avoid high penalty events and enjoy low levels of transient anxiety states. However, compared to adaptive deck selection, which comprises risk-taking with the tendency to successfully avoid losses, persistent low risk-taking may result in earning relatively small amounts of final winnings, which is potentially associated with sustained or less state anxiety due to not realizing one’s ideal final winnings. If trait anxiety is more related to sustained future-oriented anxiety during the task, people with higher trait anxiety may more frequently select high-risk decks in particular under information ambiguity without deck information, as observed in previous studies (Miu et al., 2008; Engelmann et al., 2015). On the other hand, when frequently selecting high-risk decks to obtain large gains, people can potentially attenuate sustained anxiety concerning the task mission if high penalty events do not occur. However, people may actually often face high penalty events and strongly and frequently experience transient state anxiety. Thus, people with higher state anxiety may make lower risky choice. If such a trade-off between trait and state anxiety during decision-making in the IGT can be predicted by pre-specified state and trait anxious characteristics, advance information about anxiety profiles and task performances may be useful for people to prevent future negative consequences.

The current study, therefore, assessed participants’ state and trait anxiety before the IGT and conducted the IGT under different temporal pressures to make a temporally causal prediction analysis of participants’ decision-making behaviors by anxiety profiles. We examined not only overall or global decision-making but also change in local performances immediately after salient penalty events, because coping with salient negative events under temporal pressure, such as post-error recovery, is difficult even in healthy populations (Soshi et al., 2015), although it is important to avoid subsequent negative consequences. We then treated the two disadvantageous decks separately, as suggested by a previous study (Buelow and Suhr, 2013). In the structure of the standard IGT, Deck B includes one maximum penalty card ($ -1,250) randomly ordered in each of the 10-trial blocks (i.e., 10%) (Bechara et al., 1994). The maximum penalty event, therefore, is a probabilistically rare, unpredictable negative event and is suitable for predicting future decision-making performances after saliently negative events by anxiety profiles. To examine global decision-making performances, we calculated popular behavioral indexes of risk-taking such as net scores (advantageous deck minus disadvantageous deck), final winnings, and the total number of maximum penalty events.

We hypothesized the following: (1) pre-specified trait anxiety predicts global decision-making performances differently in the self- and forced-paced conditions, because trait anxiety is likely related to sustained anxiety concerning the flaw in the final mission of the IGT (i.e., maximizing the final winnings); and (2) pre-specified state anxiety predicts local decision-making performances differently in the self- and forced-paced conditions, because state anxiety is likely related to transient sensitivity to sudden negative events, thereby instantaneously promoting risk-avoidance. Such hypothesized functions of trait and state anxiety may dynamically determine individual decision-making performances in daily social life, and the pre-specification of individual anxiety profiles is likely effective for individuals to develop a strategy for coping with future negative events in daily life.

## Materials and Methods

### Participants

The present study enrolled 33 participants [men: 19 individuals, age (mean ± SD) = 21.4 ± 2.5 years, intelligence quotient (IQ) = 109.5 ± 6.0; women: 14 individuals, age = 22.6 ± 2.0 years, IQ = 113.6 ± 3.9]. They were locally recruited through an advertisement at the Tokyo Institute of Technology and were given 2,000 yen as the baseline reward, and an optional bonus of not more than 3,000 yen depending on their task performance results. All of them had normal or corrected-to-normal visual acuity, and self-reported that they did not have any current and past psychiatric or neurological histories and had not taken medicine for any illness. Their IQ was assessed using the Japanese version of the National Adult Reading Test (Nelson and Wilson, 1991; Matsuoka and Kim, 2007). Written informed consent was obtained from the participants according to the institutional guidelines before conducting the experiment. The study was conducted in accordance with the Declaration of Helsinki, a statement of ethical principles for medical research involving human participants, and was approved by the Ethics Committee of the Tokyo Institute of Technology.

### Iowa Gambling Task

We used the IGT task developed by Bechara et al. (1994) to examine change in decision-making performances under temporal pressure. The mission of the task was to maximize the final winnings based on an initial fund of 200,000 yen. Participants selected one of the four advantageous or disadvantageous decks in each of the 100 trials with no information about the total number of trials. Frequent choice of the disadvantageous decks as risk-taking leads to smaller gains at the end of the task. Decks 1 and 2 are disadvantageous decks with constantly high returns of 10,000 yen, but randomly produce high monetary loss. Deck 1 is a deck with frequent penalties and randomly distributes five instances of monetary loss from -15,000 to -35,000 yen, with steps of 5,000 yen in each set of 10 trials, which amounts to a total penalty of -125,000 yen. Deck 2 is a deck with infrequent penalties as well as the highest penalty, randomly including one penalty of -125,000 yen in each set of 10 trials. Decks 3 and 4 are advantageous decks, with lower rewards of 5,000 yen and smaller penalties. Deck 3 frequently and randomly includes five trials of monetary loss ranging from -2,500 to -7,500 yen, with steps of 2,500 yen in each set of 10 trials with a total penalty of -25,000 yen. Deck 4 infrequently dispenses one penalty card of -25,000 yen randomly in each set of 10 trials. Because the participants in the present study performed the IGT in both the self- and forced-paced conditions, the four decks were randomly arranged so as not to appear in the same order for the two conditions. The present study used a modified version of the IGT program implemented in Cognitive Experiments V v1 (Neurobehavioral Systems, Inc., Berkeley, CA, United States) for the Japanese-translated version.

### Psychological Assessment

To investigate how pre-specified anxiety characteristics are related to subsequent decision-making behaviors in the self- and forced-paced conditions, we assessed participants’ trait and state anxiety using the Spielberger State-Trait Anxiety Inventory (STAI) (Spielberger et al., 1970; Spielberger, 1989). This scale assesses the levels of trait anxiety (STAI-T) and state anxiety (STAI-S) with 40 items; STAI-T measures the stable temperament of behaving anxiously, whereas STAI-S measures individuals’ current transient anxious status in response to emotional events and situational changes. The participants completed both scales after arriving at the experimental room and underwent an interview about 1 h before performing the IGT.

### Procedure

Participants came to a quiet experimental room to perform the IGT in the self- and forced-paced conditions. They were first provided with instructions concerning the aim and procedure of the experiment on ethical guidelines. As has been argued in the previous study (Bull et al., 2015), task-instruction manners strongly affect participants’ ability to build optimal strategies. The original task instructions include the necessary information about deck types (advantageous and disadvantageous) and behavioral preferences for avoiding disadvantageous decks to evade large monetary loss (Bechara et al., 2000b). Such rich and unambiguous information has been important for sophisticated IGT performances (Horne and Lowe, 1993; Balodis et al., 2006; Fernie and Tunney, 2006; Glicksohn and Zilberman, 2010; Bull et al., 2015). The aim of the present study, on the other hand, is to elucidate change in relationships between decision-making behaviors and pre-specified anxiety under intensified ambiguity about the task-structure information in different temporally pressured conditions. Therefore, we minimally instructed participants to maximize the initial fund of 200,000 yen without hints about deck types and a performance strategy, and maximized information ambiguity, which in turn may promote anxious behaviors under information ambiguity (Kalin and Shelton, 1989; Robinson et al., 2013), as in a previous study (Glicksohn et al., 2007). Subsequently, participants faced a 19-inch PC monitor (DELL) placed 0.65 m in front of them and completed a short practice session to learn to manipulate the response pad (RB-740, Cedrus, Corp., San Pedro, CA, United States). The practice session used dummy decks that were randomly assigned rewards and losses to possess indiscriminate patterns of card sequences for purpose to avoid guessing structural information of the decks used in the trials. After understanding the task procedure, participants performed the IGT in the self- and forced-paced conditions, which were separated by a 5-min rest interval. The task order was counterbalanced across participants. In the self-paced condition, participants were provided with sufficient time to select one of the four decks at their own pace, and the feedback display showed the current gain and loss as well as the total payoff for 2500 ms. The self-paced condition, therefore, provided participants with sufficient time to learn to discriminate between the advantageous and disadvantageous decks. The forced-paced condition, on the other hand, required participants to select a deck as soon as possible after viewing the main deck-selection display under temporal pressure. The forced-paced instruction (“Choose quickly!”) always appeared immediately below the decks at the start of each main display and remained until participants selected a deck. The feedback displays appeared for only 500 ms and were soon followed by the main display again. After completing all the tasks, participants self-assessed their deck selection patterns based on five categories: “1” = I intended to select the deck with low rewards and infrequent lower penalty (Deck 4); “2” = I intended to select the deck with low rewards and frequent lowest penalty (Deck 3); “3” = I intended to select the deck with high rewards and infrequent highest penalty (Deck 2); “4” = I intended to select the deck with high rewards and frequent higher penalties (Deck 1); and “5” = others.

### Behavioral Indexes for Decision-Making Behavior

#### Global Deck-Selection Behaviors

We first analyzed global behavioral patterns of the 100 trials overall, using two traditional indexes and eight additional indexes. The first popular index is the final winnings (yen), which indicates that higher risk-taking for disadvantageous decks leads to smaller final winnings. The second traditional index is the net scores of the five trial blocks (Block 1: trials 1–20, Block 2: 21–40, Block 3: 41–60, Block 4: 61–80, Block 5: 81–100), which are calculated by subtracting the number of the selected disadvantageous decks (Decks 1 and 2) from the number of the selected advantageous decks (Decks 3 and 4) for the index of risk-avoidance. More positive scores indicate higher preference for risk-avoidance. The third index is the number of maximum penalty events of -125,000 yen. Participants with a preference for selecting Deck 2 frequently encounter the maximum penalty events. The fourth index is the complexity of deck selection, represented by mean entropy (*H* = -Σp_i_ × log_2_p_i_; p = probability of deck choice; i = the number of the deck). Higher entropy (bit) indicates that participants more frequently change their choice of decks throughout the trials overall. For example, when participants who acquired an optimal strategy intend to avoid high risk-taking and establish larger monetary gains in their final winnings, they may tend to continuously choose advantageous decks, consequently yielding lower entropy. The fifth and sixth indexes are related to total and continuous selectivity (%) of the high-risk Deck 2. Total selectivity is the percentage of selected Deck 2 in the overall 100 trials. Continuous selectivity is the percentage of more than one continuously selected Deck 2 in the total number of selected Deck 2. These indexes indicate that higher ratios correspond to a higher preference for high risk-taking. The seventh and eighth indexes are the total and continuous selectivity of the middle-risk Deck 1, which indicates moderate risk-taking. The 9 and 10th indexes are the total and continuous selectivity of the low-risk Decks 3 and 4. Increased ratios for the low-risk decks indicate a preference for risk-avoidance, despite resulting in relatively small monetary gains in each selection but larger final winnings. Response times (RTs) for deck selection were calculated for the self- and forced-paced conditions. We first analyzed mean RTs for all 100 trials under both conditions. Second, along with the net-score analysis for temporal transition of a decision-making pattern, the overall trials were separated into the five blocks, each comprising 20 trials, and a mean RT for each block was calculated for each condition.

#### Local Deck-Selection Behaviors

**FIGURE 1:**
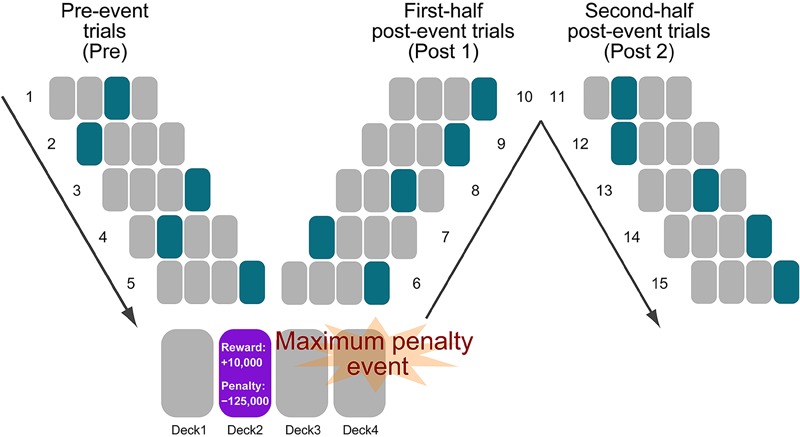
Analysis of local behavioral patterns before and after the maximum penalty event. For the purpose of elucidating local decision-making behavioral characteristics in the Iowa Gambling Task (IGT) under the self- and forced-paced conditions, pre- and post-penalty (–125,000 yen in Deck 2) trials were analyzed for each participant. First, we counted the frequency of the occurrence of the maximum penalty event for each participant. Second, by subtracting the proportions of the selected decks during the post-event trials from those during the pre-event trials, post-event changes in deck selection were examined for the high-, middle-, and low-risk decks. Pre, post 1, and post 2 indicate the five trials immediately before the event, the first-half and second-half post-event trials.

Large fluctuation in deck-selection patterns likely occurs after the maximum penalty event in Deck 2 (-125,000 yen) (Buelow and Suhr, 2013). In particular, participants with a preference for low risk-taking may tend to avoid Deck 2 and alternatively select the lower-risk decks after encountering the maximum penalty event. Therefore, we specified the occurrences of maximum penalty events and produced the indexes for local performance changes by calculating the post-event change in the selectivity of the high- (Deck 2) and low- (Decks 3 and 4) risk decks. We also examined the selection change of the middle-risk deck (Deck 1) as the index of change for moderate risk-taking. Although Deck 1 has been traditionally grouped together with Deck 2 as a disadvantageous deck, Deck 1 may be selected differently from not only high- but also low-risk decks after maximum penalty events, because it possesses the same high monetary reward of 10,000 yen and relatively low monetary loss compared to Deck 2 if not being persistently selected. We obtained individual information about the maximum penalty events until the occurrence of the third penalty event because of maintaining over 20 participants in the local performance analyses. First, we calculated the ratios of decks selected before (pre) and after (post) the maximum penalty events (Figure 1). The ratio for the pre-event trials is the percentage of selected decks in the total of the five trials immediately before the maximum penalty event. The ratios for the post-event trials consisted of two indexes. The first post-event index (post 1) is represented by the percentage of selected decks in the total of the first-half of the five post-event trials and can be used to obtain information about immediate recovery from high risk-taking. The second post-event index (post 2) is the percentage of decks occupying the five second-half trials, providing information about delayed recovery from high risk-taking. For example, participants with higher negative sensitivity to the maximum penalty might reduce post-1 ratios of the high-risk deck sooner.

### Statistical Analysis

We initially summarized participants’ self-awareness of deck-selection strategy based on their self-reported scores. We counted the numbers of participants adopting voluntary deck-selection strategies. The answers from “1” to “4” indicate that participants used their preference trend to select one of the four decks. The answer of “5” was counted as a voluntary strategy when participants self-reported that, for example, they selected decks to suppress monetary loss.

For the global performance indexes except the net score, we compared the self- and forced-paced conditions using non-parametric Wilcoxon signed-rank tests, because many index scores violated normality (Anderson-Daring tests: self-paced condition, numbers of maximum penalty events, mean entropy, continuous selectivity of high-risk decks; forced-paced condition: final winnings, numbers of maximum penalty events, mean entropy, continuous selectivity of high-risk decks, total selectivity of low-risk decks). Concerning the net score, almost all of the scores of the five blocks for the self- and forced-paced conditions also violated normality. To test the interaction effect between task (self- and forced-paced conditions) and block, we initially performed z-normalization for all of the data (5 blocks × 2 tasks × 33 participants) and conducted repeated-measures ANOVAs. Response time data for the overall 100 trials were also compared between the self- and forced-paced conditions by a paired *t*-test with normalized data. Subsequently, normalized RTs for the five blocks were analyzed with a two-way repeated-measures ANOVA with the factors of condition (self-paced, forced-paced) and block (Blocks 1–5). When a significant interaction appeared, the block effect was tested separately for each condition by follow-up ANOVAs. Over-one degrees of freedom were corrected for effects related with a trial-block factor using the Greenhouse–Geisser method (*𝜀*).

To examine the relations between global performances and anxiety characteristics in each condition, we conducted multiple linear regression analyses using each of the 10 global indexes as a dependent variable (*Y*) and STAI-T and STAI-S, which were recorded before the IGT, as independent predictive variables (*X*), controlling for age, sex, IQ, and task order (first = “1,” second = “2”). All of the independent and control variables were initially introduced into a regression model, and variables with weak coefficients were successively eliminated in a backward elimination manner. We adopted an explanatory model based on the following criteria: (i) STAI-T and/or STAI-S were significantly included (β: *p* < 0.05), and (ii) explanatory power (adjusted *R*^2^) was the highest among the significant models (*F*-value: *p* < 0.05). If significant models did not include the STAI variables, we reported the significant model with the highest explanatory power. Multicollinearity between the independent variables was examined by variance inflation factors (*VIF*s) based on the criterion that *VIF*s exceeding 10 indicate severe multicollinearity.

For the local performance indexes, we first compared the three ratios of pre- and post-event trials (pre, post 1, and post 2) with a non-parametric Friedman test. When significant trial-phase effects appeared (χ^2^: *p* < 0.05), planned Wilcoxon tests were applied between the pre and post ratios (pre vs. post 1, pre vs. post 2). Multiple regression analyses were also performed to examine the relation between post-event performance changes and anxiety characteristics in a similar manner as in the global performance analysis. The model used the subtraction ratios between pre- and post-event trials (post 1 or post 2 minus pre) as the dependent variable (*Y*), STAI-T and STAI-S before the IGT as the independent variable (*X*), controlling for age, sex, IQ, and task order. We reported significant models based on the criterion adopted in the global performance analysis.

Non-parametric tests for the local performance indexes and regression analyses for both the global and local performances were multiply conducted (non-parametric tests: 3 post-penalty phases × 3 deck types × 2 task conditions = 18 analyses; regression analysis: global, 9 performance indexes × 2 task conditions = 18 analyses; local: 2 post-penalty phases × 3 error events × 3 deck types × 2 task conditions = 36 analyses). This analysis condition allows us to take into consideration both type I errors concerning overestimation of significant explaining models and type II errors involving underestimation of significant effects under intensive correction. Accordingly, to examine the reliabilities of the observed effects under the multiple-testing correction, we performed permutation tests in which samples were randomly and multiply resampled from the original data, and the original results were tested using a *post hoc* permutation distribution of dummy outputs (Nichols and Holmes, 2002), based on the notion that overestimation of significant effects from multiple testing was avoided by data-driven thresholds obtained from at-issue multiple tests.

In the non-parametric permutation tests for the local performance, the raw data of the three trial phases (pre, post 1, post 2) at the maximum penalty events (1st, 2nd, 3rd) in the three deck types (high, middle, low) were transformed into *z*-scores across participants and combined into a single data set (*n* = 1,188). All data were randomly reordered and same numbers of samples with participants were chosen for each trial phase (e.g., self-paced: *n* = 32 for the first post-error phase, *n* = 30 for the second post-error phase, or *n* = 24 for the third post-error phase) for Friedman tests. This resampling procedure was repeated 100,000 times with different sample sizes for each penalty event in the two task conditions to obtain dummy *p*-values. When actual *p*-values in the original tests (3 penalty events × 3 deck types × 2 task conditions = 18) were within the lower 5% range of the distribution of 100,000 dummy *p*-values, they were certified as significance-corrected for multiple testing. Similarly, *post hoc* Wilcoxon signed-rank tests used a permutation method to determine a *p*-value threshold for each pairwise comparison.

In the regression analyses, a similar permutation method was performed as follows: each dependent variable (e.g., final winnings in the global analysis) in the self- and forced-paced conditions was transformed into *z*-scores across participants. Transformed variables were combined into a single data set separately for each performance analysis, because global and local performance analyses included different dependent variables. Integrated data were randomly reordered and the same numbers of samples as the participants were chosen as a dummy dependent variable (global analysis: *n* = 33; local analysis for, e.g., the self-paced condition: *n* = 32 for the first penalty event; *n* = 30 for the second penalty event; *n* = 24 for the third penalty event), being regressed by original independent variables including STAI-S and/or STAI-T. Each resampling test was conducted 100,000 times to produce a distribution of dummy *p*-values. When original *p*-values were lower than the *p*-value thresholds (5% borders of the dummy *p*-value distributions), they were considered significant under correction for multiple testing.

## Results

### Participants’ Voluntary Deck-Selection Strategy

Among the 33 participants, 29 (88%) adopted voluntary strategies. Nine participants answered “1” for Deck 4; four, “2” for Deck 3; seven, “3” for Deck 2; and three, “4” for Deck 1. Among the 10 participants who answered “5,” six self-reported using their own strategies. This suggests that the participants felt that they executed the IGT while developing and controlling individual decision-making strategies.

### Global Task Behaviors

We initially compared the nine deck-selection indexes except the net score between the self- and forced-paced conditions using the Wilcoxon signed-rank test. No task performances yielded significant differences (final winnings: *Z* = 0.617, *p* = 0.537; penalty events: *Z* = 0.061, *p* = 0.951; entropy: *Z* = 0.524, *p* = 0.600; high-risk deck: total selectivity, *Z* = 0.804, *p* = 0.422 and continuous selectivity, *Z* = 0.253, *p* = 0.801; middle-risk deck: total selectivity, *Z* = 0.175, *p* = 0.861 and continuous selectivity, *Z* = 0.170, *p* = 0.865; low-risk deck: total selectivity, *Z* = 0.751, *p* = 0.453 and continuous selectivity, *Z* = 1.108, *p* = 0.268) (Supplementary Table S1).

**FIGURE 2:**
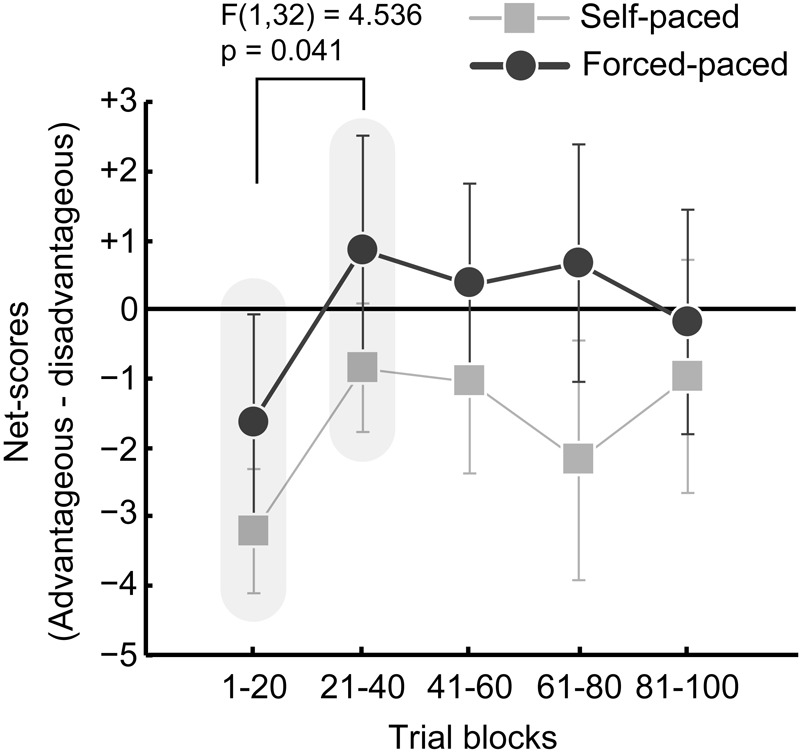
Change in advantageous deck selection patterns in the self- (gray) and forced-paced (black) conditions. The net score for each set of 20 trials was calculated by subtracting the numbers of selected disadvantageous decks (Deck 1 + Deck 2) from the numbers of selected advantageous decks. Larger scores indicate more frequent risk-avoidance. An ANOVA with the factors of condition (self-paced, forced-paced) and block (Block 1, Block 2) showed that net scores significantly increased in the second block in both conditions. Error bars indicate standard errors of the means.

The results of the net scores are plotted in Figure 2. The mean net scores with standard errors of the means are -3.1 ± 0.9, -0.8 ± 0.9, -0.9 ± 1.3, -2.1 ± 1.7, and -0.9 ± 1.7 for the five blocks in the self-paced condition, and -1.5 ± 1.6, 0.9 ± 1.7, 0.5 ± 1.4, 0.8 ± 1.7, and -0.1 ± 1.6 for the five blocks in the forced-paced condition, respectively. The initial ANOVA with normalized data did not indicate a significant difference between the two conditions [condition: *F*(1,32) = 0.988, *p* = 0.328; block: *F*(4,128) = 1.275, *p* = 0.287; condition × block: *F*(4,128) = 0.267, *p* = 0.899]. Although the lack of a significant effect may be surprising in light of previous findings, it is suspected that the current experimental setting increased individual variation in transitions of deck-selection patterns by minimizing task-related instruction without hints about deck types and an optimal deck-selection strategy. Accordingly, we made planned comparisons between Block 1 and Block 2 as a phase transition from exploratory or pre-hunch to predictive phases (Bechara et al., 2000a): actually, net scores changed from Block 1 to Block 2 based on visual inspection. A two-way ANOVA observed a significant block effect across the two task conditions [block: *F*(1,32) = 4.536, *p* = 0.041, $\eta_{p}^{2}$ = 0.124; condition: *F*(1,32) = 1.067, *p* = 0.309; condition × block: *F*(1,32) = 0.003, *p* = 0.955], indicating that the participants reduced their risk-taking proportions after the initial exploring phase in both task conditions.

**FIGURE 3:**
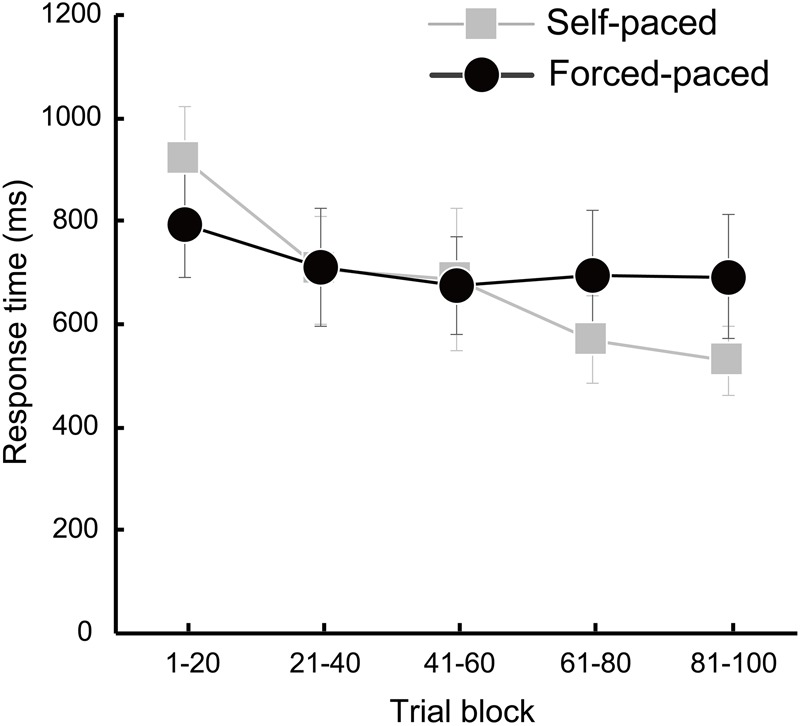
Changes in deck-selection speeds throughout the five trial blocks in the self- (gray) and forced-paced (black) conditions. Mean response time (RT) for each trial block was calculated for the 20 trials in each task condition. A repeated-measures ANOVA was conducted with the factors of condition and block. A significant block effect was observed only in the self-paced condition (*p* < 0.0001).

Response times for all trials in the self- and forced-paced conditions are 683 ± 92 and 713 ± 110 ms, respectively and were not significantly different in a paired *t*-test [*t*(32) = 0.550, *p* = 0.586]. Response times for the five blocks in the self- and forced-paced conditions are summarized in order as 921 ± 102, 706 ± 105, 687 ± 139, 571 ± 85, and 530 ± 68 ms for the self-paced condition, and 793 ± 102, 711 ± 115, 676 ± 94, 694 ± 128, and 692 ± 120 ms for the forced-paced condition. The summary suggests that the self-paced condition but not the forced-paced condition reduced RTs as progression of the trials (Figure 3), which was statistically confirmed. The initial two-way ANOVA indicated the significant interaction of condition × block [*F*(4,128) = 3.762, *p* = 0.023, $\eta_{p}^{2}$ = 0.105, *𝜀* = 0.569]. The self-paced condition yielded a significant block effect [*F*(4,128) = 9.979, *p*< 0.0001, $\eta_{p}^{2}$ = 0.238, *𝜀* = 0.628], and the later blocks significantly decreased RTs over the initial block in the planned comparison [Block 1 vs. Block 5: *p* < 0.0001]. The forced-paced condition, on the other hand, did not show a significant block effect [*F*(4,128) = 2.811, *p* = 0.060, *𝜀* = 0.566], which indicates that decision-making speed did not alter throughout the overall trials.

**FIGURE 4:**
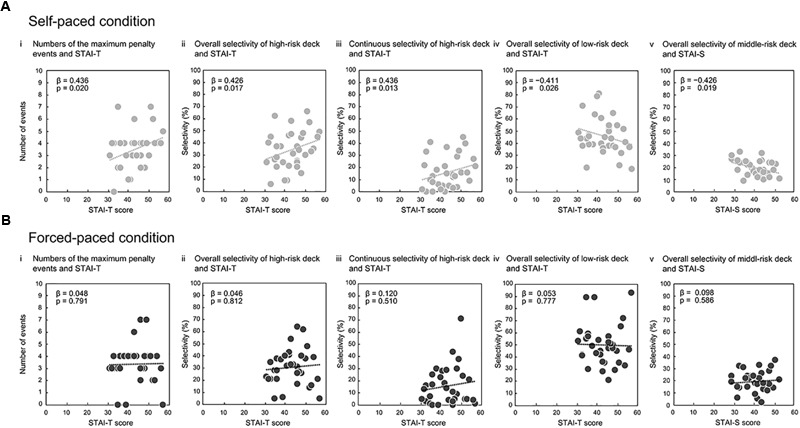
Comparisons of linear relations between global behavioral patterns and trait anxiety between the self- and forced-paced conditions. Scatter plots for the self-paced condition represent significant relations between: the numbers of the maximum penalty events and trait anxiety (STAI-T) **(Ai)**; overall high-risk deck selectivity and STAI-T **(Aii)**; continuous high-risk deck selectivity and STAI-T **(Aiii)**; and overall low-risk deck selectivity and STAI-T **(Aiv)**. The total selectivity of the middle-risk deck was predicted by state anxiety (STAI-S) **(Av)**. Corresponding figures **(Bi–v)** for the forced-paced condition show no significant relation between behavioral patterns and STAI-T. Weighted coefficients (βs) indicate standardized partial coefficients in the multiple regression models.

The self- and forced-paced conditions showed different relational characteristics between global deck-selection performances and anxiety characteristics. In the self-paced condition, four deck-selection characteristics for risk-taking were significantly predicted by STAI-T (Table 1). The numbers of maximum penalty events (Figure 4Ai), total and continuous selectivity of the high-risk deck (Figure 4Aii,iii), and total selectivity of the low-risk deck (Figure 4Aiv) were significantly predicted by STAI-T (maximum penalty events: β = 0.436, *t* = 2.460, *p* = 0.020, *VIF* = 1.153; high-risk deck: total selectivity, β = 0.426, *t* = 2.541, *p* = 0.017, *VIF* = 1.156, and continuous selectivity, β = 0.436, *t* = 2.636, *p* = 0.013, *VIF* = 1.155; low-risk deck: total selectivity, β = -0.411, *t* = 2.242, *p* = 0.033, *VIF* = 1.270). That is, higher anxiety traits yielded more penalty events,more frequent selection of the high-risk deck, and less frequent selection of the low-risk decks. The total selectivity of the middle-risk deck was predicted by STAI-S (β = -0.426, *t* = 2.468, *p* = 0.019, *VIF* = 1.101) (Figure 4Av). However, in the forced-paced condition (Table 2), no deck-selection property was sig-nificantly predicted by either STAI-T or STAI-S (Figure 4Bi–v). The permutation tests for correction in multiple testing confirmed the observed results: actual *p*-values of the significant models were below the accidental-level *p*-value thresholds, set as the border of the lower 5% of dummy *p*-values (see, for example, Supplementary Figure S1) in both the self-paced condition [model with STAI-T (*p*-value threshold = 0.0506): maximum penalty events, *p* = 0.049; total selectivity in the high-risk deck, *p* = 0.025; total selectivity in the low-risk deck, *p* = 0.048; model with STAI-T and Age (*p*-value threshold = 0.0503): continuous selectivity in the high-risk deck, *p* = 0.012; model with STAI-S (*p*-value threshold = 0.0509): total selectivity in the middle-risk deck, *p* = 0.044].

**Table 1:** Regression models of overall behavioral patterns in the self-paced condition (*n* = 33).

	Overall (100 trials)						High-risk deck			
			Numbers of maximum	
Variables	Final winning		penalty events		Mean entropy		Total selectivity		Continuous selectivity	
	β	*t*-Value (*p*-value)	β	*t*-Value (*p*-value)	β	*t*-Value (*p*-value)	β	*t*-Value (*p*-value)	β	*t*-Value (*p*-value)
STAI-T	-0.351	1.834 (0.077)	0.436*	2.460 (0.020)	0.149	0.797 (0.432)	0.426*	2.541 (0.017)	0.436*	2.636 (0.013)
STAI-S	0.300	1.580 (0.125)	-0.104	0.544 (0.591)	-0.224	1.350 (0.187)	-0.075	0.414 (0.682)	0.019	0.106 (0.916)
Age	-0.179	0.946 (0.352)	0.291	1.641 (0.111)	-0.095	0.508 (0.615)	0.317	1.747 (0.092)	0.381*	2.143 (0.041)
Sex	-0.208	1.223 (0.231)	0.203	1.199 (0.240)	-0.071	0.394 (0.696)	0.194	1.145 (0.262)	0.155	0.923 (0.364)
IQ	-0.105	0.568 (0.574)	0.145	0.807 (0.426)	-0.398*	2.401 (0.023)	0.327	1.845 (0.076)	0.229	1.371 (0.181)
Task order	-0.045	0.263 (0.795)	0.072	0.429 (0.671)	-0.136	0.807 (0.426)	0.146	0.905 (0.374)	0.108	0.673 (0.506)
Regression model
Adjusted *R*^2^	0.089		0.128		0.133		0.221		0.241	
*F*-value	2.043		3.346		3.458		3.272		4.384	
*p*-value	0.13		0.049^∗^		0.045^∗^		0.025^∗^		0.012^∗^	

	Middle-risk deck				Low-risk deck			
Variables	Total selectivity		Continuous selectivity		Total selectivity		Continuous selectivity	
	β	*t*-Value (*p*-value)	β	*t*-Value (*p*-value)	β	*t*-Value (*p*-value)	β	*t*-Value (*p*-value)
STAI-T	-0.071	0.363 (0.719)	0.025	0.142 (0.888)	-0.411*	2.350 (0.026)	-0.042	0.253 (0.802)
STAI-S	-0.426*	2.468 (0.019)	-0.022	0.128 (0.899)	0.293	1.598 (0.121)	0.184	1.124 (0.270)
Age	-0.281	1.628 (0.114)	0.069	0.365 (0.718)	-0.153	0.919 (0.366)	0.134	0.743 (0.463)
Sex	0.007	0.041 (0.968)	-0.271	1.481 (0.149)	-0.170	0.967 (0.342)	-0.315	1.792 (0.083)
IQ	-0.044	0.242 (0.811)	-0.353	1.928 (0.063)	-0.309	1.888 (0.069)	-0.448*	2.549 (0.016)
Task order	0.034	0.201 (0.842)	-0.174	1.000 (0.325)	-0.153	0.919 (0.366)	-0.151	0.902 (0.375)
Regression model
Adjusted *R*^2^	0.134		0.07		0.155		0.143	
*F*-value	3.473		2.205		2.963		3.673	
*p*-value	0.044^∗^		0.128		0.048^∗^		0.037^∗^	


*^∗^*p*< 0.05.*

**Table 2:** Regression models of overall behavioral patterns in the forced-paced condition (*n* = 33).

	Overall (100 trials)						High-risk deck			
			Numbers of maximum	
Variables	Final winning		penalty events		Mean entropy		Total selectivity		Continuous selectivity	
	β	*t*-Value (*p*-value)	β	*t*-Value (*p*-value)	β	*t*-Value (*p*-value)	β	*t*-Value (*p*-value)	β	*t*-Value (*p*-value)
STAI-T	0.137	0.760 (0.454)	0.048	0.268 (0.791)	-0.195	1.219 (0.233)	0.046	0.240 (0.812)	0.120	0.666 (0.510)
STAI-S	0.030	0.171 (0.866)	0.019	0.108 (0.915)	0.016	0.086 (0.932)	-0.045	0.239 (0.812)	0.018	0.102 (0.920)
Age	0.201	1.108 (0.277)	-0.159	0.840 (0.407)	-0.019	0.100 (0.921)	-0.196	1.035 (0.309)	-0.137	0.746 (0.462)
Sex	0.226	1.259 (0.218)	0.018	0.092 (0.927)	-0.267	1.569 (0.128)	0.007	0.038 (0.970)	0.179	1.012 (0.319)
IQ	-0.340	-1.772 (0.087)	0.247	1.420 (0.166)	0.232	1.340 (0.191)	0.293	1.550 (0.132)	0.035	0.183 (0.856)
Task order	0.299	1.766 (0.088)	-0.151	0.847 (0.404)	-0.352*	2.181 (0.038)	-0.088	0.485 (0.631)	0.097	0.543 (0.591)
Regression model
Adjusted *R*^2^	0.123		0.031		0.202		0.019		0.001	
*F*-value	2.125		2.015		3.024		1.317		1.024	
*p*-value	0.104		0.166		0.034^∗^		0.283		0.319	

	Middle-risk deck						Low-risk deck			
Variables	Total selectivity		Continuous selectivity		Total selectivity		Continuous selectivity	
	β	*t*-Value (*p*-value)	β	*t*-Value (*p*-value)	β	*t*-Value (*p*-value)	β	*t*-Value (*p*-value)
STAI-T	-0.198	1.196 (0.242)	-0.176	0.896 (0.377)	0.053	0.286 (0.777)	0.027	0.155 (0.878)
STAI-S	0.098	0.551 (0.586)	0.226	1.304 (0.202)	0.023	0.127 (0.900)	0.156	0.917 (0.367)
Age	-0.345	1.922 (0.065)	0.091	0.494 (0.625)	0.298	1.633 (0.114)	0.267	1.524 (0.138)
Sex	-0.446*	2.684 (0.012)	-0.139	0.798 (0.431)	0.218	1.204 (0.239)	0.131	0.739 (0.466)
IQ	0.211	1.187 (0.246)	0.022	0.121 (0.904)	-0.354	1.834 (0.077)	-0.492**	-2.810 (0.009)
Task order	-0.319	2.024 (0.053)	-0.218	1.259 (0.218)	0.228	1.337 (0.192)	0.027	0.158 (0.875)
Regression model
Adjusted *R*^2^	0.25		0.04		0.11		0.161	
*F*-value	3.129		1.661		1.993		4.069	
*p*-value	0.024^∗^		0.207		0.123		0.027	


*^∗^*p*< 0.05; ^∗∗^*p*< 0.01.*

### Local Task Behaviors

We specified three earlier maximum penalty events (-125,000 yen) and locally examined post-penalty behaviors. We first calculated the selection rates of the high-, middle-, and low-risk decks immediately before and after the maximum penalty event separately for the self- and forced-paced conditions and compared them using Friedman tests. We then calculated indexes for post-event behavioral change by subtracting the pre-event rates from post-event rates. The difference rate scores, as the dependent variable, were introduced into a regression analysis and were predicted by the independent variables of STAI-T and STAI-S before the IGT.

**FIGURE 5:**
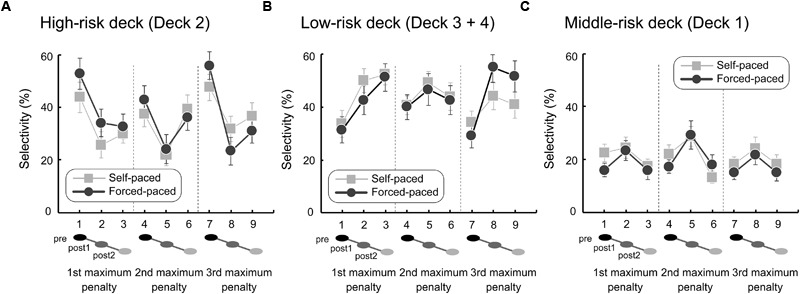
Local behavioral patterns of high, middle, and low risk-taking in the self- and forced-paced conditions. We locally examined the high-risk-taking behavioral characteristics immediately before and after the maximum penalty event (–125,000 yen in Deck 2). The high-risk-taking property **(A)** was calculated with the pre- and first-half (post 1) and second-half (post 2) post-event proportions of Deck 2 selection for the total of each five-trial interval. The low-risk-taking property **(B)** was also calculated for the combined Decks 3 and 4. The middle-risk-taking property **(C)** was similarly calculated for Deck 1. Error bars indicate standard errors of the means.

As observed in Figure 5, both the self- and forced-paced conditions showed similar deck-selection trends for the three types of decks. Selection of the high-risk deck generally decreased after the maximum penalty events (post 1, post 2) (Figure 5A). Conversely, selection of the low-risk decks tended to increase after the penalty events in both the self- and forced-paced conditions (Figure 5B). Although the middle-risk deck has been generally grouped with Deck 2 as a disadvantageous deck, the selectivity showed a flat pattern throughout the overall intervals (Figure 5C) that somewhat differed from the selection pattern of the high-risk deck (3 pre and post intervals × 3 penalty events = 9 points: *r* = -0.63) but was relatively similar to that of the low-risk decks (*r* = 0.31).

Non-parametric statistical tests revealed post-penalty behavioral changes in the self-paced condition (Supplementary Table S2). Selectivity changes were significant for the first penalty in the low-risk deck and the second penalty in the high- and middle-risk decks in the Freidman tests [low-risk: χ^2^ = 9.05, *p* = 0.011; high-risk: χ^2^ = 7.22, *p* = 0.027; middle-risk: χ^2^ = 10.02, *p* = 0.007]. These *p*-values were below the *p*-value thresholds (the border of the lower 5% *p*-value distributions) in the permutation tests [1st (*n* = 32): *p*-value threshold = 0.0543; 2nd (*n* = 30): *p*-value threshold = 0.0482] and were certified as significant after correction for multiple testing. In multiple comparisons, the selection of the high-risk deck significantly decreased during the post-1 trials after the second penalty event [Wilcoxon test: pre (37.3%) vs. post 1 (22.0%), *Z* = 2.331, *p* = 0.020 (<*p*-value threshold, 0.0504)]. Safe-preference after the penalty event was also observed for the low-risk decks: after the first maximum-penalty, selectivity significantly increased during both the post-1 and post-2 trials [pre (33.8%) vs. post 1 (50.0%): *Z* = 2.545, *p* = 0.011; pre vs. post 2 (52.5%): *Z* = 2.911, *p* = 0.004; (<*p*-value threshold, 0.0518)]. On the other hand, the selectivity of the middle-risk deck might have been affected by the increase in the selectivity of the high-risk deck, decreasing after the second penalty event [pre (22.0%) vs. post 2 (13.3%): *Z* = 2.124, *p* = 0.034 (<*p*-value threshold, 0.0504)].

For the forced-paced condition, similar post-penalty preference for risk-avoidance was observed in the selection behaviors for the high- and low-risk decks (Supplementary Table S3). Friedman tests showed that main effects of selectivity were significant for the second penalty in the high-risk deck and the third penalty in the high- and low-risk decks (high-risk: 2nd, χ^2^ = 7.67, *p* = 0.022; 3rd, χ^2^ = 11.61, *p* = 0.003; low-risk: 3rd, χ^2^ = 9.07, *p* = 0.011). These *p*-values were below the *p*-value threshold [2nd (*n* = 30): *p* = 0.0482; 3rd (*n* = 24): *p* = 0.0478] and were certified as corrected for multiple analyses. In subsequent Wilcoxon tests, the selectivity of the high-risk deck significantly decreased [2nd: pre (42.7%) vs. post 1 (24.0%): *Z* = 2.502, *p* = 0.012; 3rd: pre (55.8%) vs. post 1 (23.3%), *Z* = 3.090, *p* = 0.002; pre vs. post 2 (30.8%), *Z* = 2.653, *p* = 0.008]. The selectivity of the low-risk deck significantly increased after the third penalty event [pre (29.2%) vs. post 1 (55.0%): *Z* = 2.666, *p* = 0.008; pre vs. post 2 (51.7%): *Z* = 2.388, *p* = 0.017]. The observed *p*-values were lower than the *p*-value thresholds in the permutation tests (2nd: *p* = 0.0504; 3rd: *p* = 0.0520) and were certified as significant under correction for multiple testing. The selectivity of the middle-risk deck, on the other hand, did not show any significant change.

**FIGURE 6:**
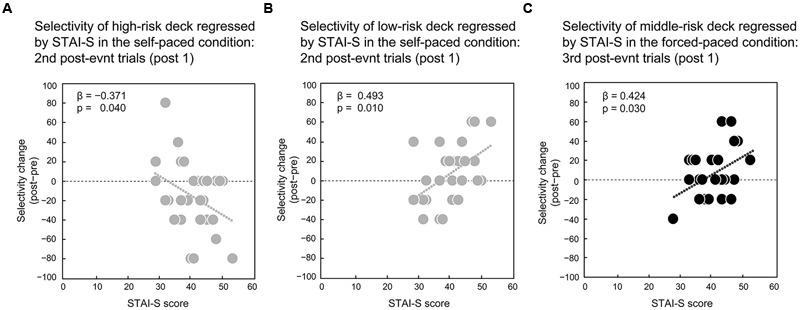
Linear relations between deck selectivity after the maximum penalty events and mood characteristics for the self- and forced-paced conditions. Change in high-risk-deck selectivity after the second maximum penalty was negatively correlated with state anxiety (STAI-S) in the self-paced condition **(A)**. Change in low-risk-deck selectivity after the second maximum penalty event was positively correlated with STAI-S in the self-paced condition **(B)**. Change in middle-risk-deck selectivity after the third maximum penalty event was positively correlated with STAI-S in the forced-paced condition **(C)**. Post-event selectivity change was calculated by subtracting pre-event proportions of the given deck from post-event counterparts. Post 1 and post 2 indicate the first-half and second-half post-event trials, respectively.

Unlike global decision-making behaviors, local behaviors were well-predicted by STAI-S in the multiple regression analyses. The self- and forced-paced conditions showed contrasted outputs. For the self-paced condition, STAI-S significantly predicted risk-avoidance behaviors (Table 3). Although the regression model for the post-1 trials after the second penalty event tended to be significant [adjusted *R*^2^ = 0.171, *F*(4,25) = 2.493, *p* = 0.069], higher STAI-S scores predicted more frequent avoidance of the high-risk deck in the model (β = -0.371, *t* = 2.171, *p* = 0.040, *VIF* = 1.019). Higher STAI-S also predicted higher preference for the low-risk deck during the post-1 trials after the second penalty event [adjusted *R*^2^ = 0.218, *F*(3,26) = 3.691, *p* = 0.024; STAI-S: β = 0.493, *t* = 2.786, *p* = 0.010, *VIF* = 1.159] (Figure 6A,B). During the post-1 trials after the third penalty event, higher STAI-T predicted higher frequent avoidance of the high-risk deck [adjusted *R*^2^ = 0.311, *F*(4,19) = 3.592, *p* = 0.024; STAI-T: β = -0.525, *t* = 2.308, *p* = 0.032, *VIF* = 1.729]. The permutation tests showed that the observed *p*-values for the significant models were below the *p*-value thresholds [model with STAI-S (*p*-value threshold = 0.0497): post 1 after the second penalty in the low-risk deck, *p* = 0.024; model with STAI-T (*p*-value threshold = 0.0504): post 1 after the third penalty in the high-risk deck, *p* = 0.024].

**Table 3:** Regression models of local deck-selection changes (post minus pre) after maximum penalty events in the self-paced condition.

	Selectivity changes (post minus pre)											
	1st (*n* = 32)				2nd (*n* = 30)				3rd (*n* = 24)			
	Post 1		Post 2		Post 1		Post 2		Post 1		Post 2	
		*t*-Value		*t*-Value		*t*-Value		*t*-Value		*t*-Value		*t*-Value
	β	(*p*-value)	β	(*p*-value)	β	(*p*-value)	β	(*p*-value)	β	(*p*-value)	β	(*p*-value)
**High-risk deck**			
STAI-T	0.049	0.266 (0.792)	0.06	0.339 (0.737)	-0.130	0.605 (0.551)	-0.098	0.516 (0.610)	-0.525*	2.308 (0.032)	-0.055	0.266 (0.793)
STAI-S	-0.141	0.778 (0.443)	-0.272	1.659 (0.108)	-0.371*	2.171 (0.040)	0.004	0.023 (0.982)	0.482	2.075 (0.052)	-0.098	0.476 (0.639)
Age	-0.132	0.728 (0.472)	-0.144	0.765 (0.451)	-0.192	0.960 (0.347)	0.324	1.863 (0.073)	0.457*	2.314 (0.032)	0.166	0.816 (0.423)
Sex	-0.114	0.628 (0.535)	-0.131	0.738 (0.467)	-0.261	1.408 (0.171)	-0.127	0.688 (0.497)	0.416*	2.209 (0.040)	-0.173	0.852 (0.404)
IQ	-0.053	0.289 (0.774)	-0.259	1.555 (0.131)	-0.418*	2.189 (0.038)	0.003	0.016 (0.988)	-0.021	0.095 (0.925)	-0.084	0.399 (0.694)
Task order	-0.107	0.587 (0.562)	-0.342	2.070 (0.048	0.226	1.302 (0.205)	-0.279	1.605 (0.120)	-0.090	0.510 (0.616)	0.315	1.558 (0.133)
**Middle-risk deck**			
STAI-T	-0.296	1.425 (0.166)	-0.086	0.387 (0.702)	0.113	0.641 (0.527)	0.056	0.300 (0.766)	0.357	1.406 (0.176)	-0.025	0.116 (0.909)
STAI-S	0.349	1.681 (0.104)	0.194	1.083 (0.288)	0.021	0.014 (0.989)	0.119	0.648 (0.523)	-0.494	1.907 (0.072)	-0.064	0.302 (0.766)
Age	0.040	0.198 (0.845)	0.073	0.355 (0.726)	-0.011	0.115 (0.909)	-0.270	1.455 (0.158)	-0.415	1.882 (0.075)	0.000	0.000 (1.000)
Sex	-0.033	0.180 (0.859)	0.072	0.369 (0.715)	0.360	1.899 (0.068)	0.267	1.423 (0.167)	-0.373	1.722 (0.092)	-0.131	0.618 (0.543)
IQ	0.350	2.025 (0.053)	0.240	1.341 (0.190)	0.430	2.270 (0.031)	0.548**	2.841 (0.009)	-0.095	0.395 (0.698)	0.062	0.290 (0.774)
Task order	-0.236	1.376 (0.180)	0.091	0.494 (0.625)	0.026	0.142 (0.888)	0.054	0.304 (0.763)	-0.010	0.051 (0.960)	0.062	0.289 (0.775)
**Low-risk deck**			
STAI-T	0.055	0.324 (0.749)	0.077	0.446 (0.659)	0.049	0.227 (0.822)	0.213	1.175 (0.250)	0.231	1.115 (0.277)	0.139	0.661 (0.516)
STAI-S	0.030	0.176 (0.862)	0.165	0.967 (0.341)	0.493**	2.786 (0.010)	-0.034	0.147 (0.885)	0.135	0.640 (0.529)	0.189	0.904 (0.376)
Age	0.103	0.591 (0.559)	0.076	0.439 (0.664)	0.232	1.312 (0.201)	-0.175	0.835 (0.411)	-0.145	0.686 (0.500)	-0.190	0.906 (0.375)
Sex	0.299	1.789 (0.084)	0.053	0.306 (0.762)	-0.012	0.069 (0.946)	0.187	0.944 (0.354)	-0.057	0.267 (0.792)	0.097	0.457 (0.652)
IQ	-0.212	1.156 (0.257)	0.125	0.719 (0.478)	0.004	0.022 (0.983)	-0.256	1.412 (0.170)	0.078	0.369 (0.715)	0.155	0.734 (0.471)
Task order	0.318	1.903 (0.067)	0.364*	2.139 (0.041)	-0.271	1.651 (0.111)	0.180	0.970 (0.341)	0.107	0.506 (0.618)	0.014	0.066 (0.948)

*1st: the first maximum penalty event; 2nd: the second maximum penalty event; 3rd: the third maximum penalty event; pre: five trials before the penalty event; post 1: the first-half five trials after the penalty event; post 2: the second-half five trials after the penalty event; ^∗^*p*< 0.05; ^∗∗^*p*< 0.01.*

In the forced-paced condition, STAI-S significantly predicted only the selectivity of the middle-risk deck [1st: post 1, adjusted *R*^2^ = 0.118, *F*(1,28) = 4.885, *p* = 0.035; 3rd: post 1, adjusted *R*^2^ = 0.142, *F*(1,22) = 4.813, *p* = 0.039; post 2: adjusted *R*^2^ = 0.138, *F*(1,22) = 4.683, *p* = 0.042] (Table 4). Higher STAI-S scores predicted higher selectivity of the middle-risk deck (1st: post 1, β = 0.385, *t* = 2.210, *p* = 0.035, *VIF* = 1.0; 3rd: post 1, β = 0.424, *t* = 2.194, *p* = 0.039, *VIF* = 1.0; 3rd: post 2: β = 0.419, *t* = 2.164, *p* = 0.042, *VIF* = 1.0) (Figure 6C for the post-1 trials at the third penalty event). The permutation tests showed that the *p*-values for the significant models were below the thresholds of corrected *p*-values [model with STAI-S for the first penalty (*p*-value threshold = 0.0502) and the third penalty (0.0499): 1st: post 1, *p* = 0.035; 3rd: post 1, *p* = 0.039; post 2, *p* = 0.042]. These results suggest that pre-specified state anxiety is related to intermediate risk-taking in the forced-paced condition.

**Table 4:** Regression models of local deck-selection changes (post minus pre) after maximum penalty events in the forced-paced condition.

	Selectivity changes (post minus pre)											
	1st (*n* = 30)				2nd (*n* = 30)				3rd (*n* = 24)			
	Post 1		Post 2		Post 1		Post 2		Post 1		Post 2	
		*t*-Value		*t*-Value		*t*-Value		*t*-Value		*t*-Value		*t*-Value
	β	(*p*-value)	β	(*p*-value)	β	(*p*-value)	β	(*p*-value)	β	(*p*-value)	β	(*p*-value)
**High-risk deck**			
STAI-T	0.107	0.545 (0.590)	0.121	0.627 (0.536)	-0.219	1.179 (0.249)	-0.156	0.815 (0.422)	-0.307	1.412 (0.174)	-0.020	0.096 (0.924)
STAI-S	-0.297	1.702 (0.100)	-0.256	1.493 (0.147)	0.122	0.616 (0.544)	-0.088	0.469 (0.643)	-0.375	1.882 (0.075)	-0.050	0.244 (0.809)
Age	-0.173	-0.929 (0.361)	0.119	0.646 (0.524)	-0.379	1.978 (0.059)	-0.322	1.698 (0.101)	-0.317	1.523 (0.144)	-0.093	0.438 (0.666)
Sex	-0.296	1.700 (0.101)	-0.366*	2.130 (0.042)	-0.225	1.212 (0.237)	-0.221	1.167 (0.253)	-0.002	0.011 (0.991)	0.292	1.481 (0.154)
IQ	-0.014	0.073 (0.942)	0.162	0.883 (0.385)	-0.136	0.650 (0.522)	-0.158	0.777 (0.444)	0.023	-0.106 (0.917)	0.042	0.182 (0.857)
Task order	0.066	0.367 (0.717)	0.128	0.734 (0.469)	0.215	1.200 (0.241)	0.149	0.805 (0.428)	0.369	2.035 (0.056)	0.314	1.592 (0.126)
**Middle-risk deck**			
STAI-T	0.072	0.367 (0.717)	-0.308	1.552 (0.133)	-0.051	0.243 (0.810)	-0.394*	-2.145 (0.041)	-0.166	0.777 (0.446)	0.093	0.429 (0.672)
STAI-S	0.385*	2.210 (0.035)	0.207	1.048 (0.304)	-0.209	1.118 (0.273)	0.345	1.892 (0.070)	0.424*	2.194 (0.039)	0.419*	2.164 (0.042)
Age	0.064	0.356 (0.725)	-0.118	0.621 (0.540)	0.016	0.078 (0.938)	0.136	0.746 (0.463)	0.176	0.866 (0.396)	-0.066	0.317 (0.754)
Sex	-0.005	0.028 (0.978)	0.169	0.927 (0.363)	-0.183	0.927 (0.362)	-0.329	1.978 (0.059)	-0.219	1.137 (0.268)	-0.043	0.215 (0.832)
IQ	0.061	0.230 (0.820)	-0.099	0.512 (0.613)	-0.194	1.039 (0.308)	0.012	0.068 (0.947)	0.174	0.898 (0.380)	0.154	0.790 (0.439)
Task order	0.078	0.439 (0.664)	-0.332	1.846 (0.076)	0.113	0.572 (0.572)	0.107	0.631 (0.534)	0.102	0.519 (0.609)	0.076	0.388 (0.702)
**Low-risk deck**			
STAI-T	-0.087	0.465 (0.646)	0.020	0.101 (0.920)	0.280	1.607 (0.121)	0.306	1.534 (0.138)	0.291	1.543 (0.138)	0.087	0.150 (0.882)
STAI-S	0.051	0.272 (0.788)	0.242	1.369 (0.182)	0.052	0.276 (0.785)	-0.304	1.564 (0.130)	0.028	0.132 (0.896)	-0.052	0.406 (0.689)
Age	0.098	0.506 (0.617)	-0.134	0.702 (0.489)	0.298	1.567 (0.130)	0.281	1.470 (0.154)	0.235	1.095 (0.287)	0.210	1.083 (0.292)
Sex	0.261	1.428 (0.164)	0.304	1.717 (0.097)	0.349	1.955 (0.062)	0.307	1.675 (0.106)	0.053	0.275 (0.786)	-0.363	1.876 (0.075)
IQ	-0.030	0.153 (0.879)	-0.118	0.616 (0.543)	0.320	1.651 (0.112)	0.085	0.425 (0.675)	0.013	0.064 (0.949)	-0.055	0.242 (0.811)
Task order	-0.098	0.525 (0.604)	0.007	0.039 (0.969)	-0.290	1.644 (0.113)	-0.119	0.657 (0.518)	-0.417*	2.213 (0.038)	-0.329	1.786 (0.089)

*1st: the first maximum penalty event; 2nd: the second maximum penalty event; 3rd: the third maximum penalty event; pre: five trials before the penalty event; post 1: the first-half five trials after the penalty event; post 2: the second-half five trials after the penalty event; ^∗^*p*< 0.05.*

## Discussion

The present study conducted a decision-making experiment using the IGT to examine how trait and state anxiety profiles predict future decision-making performances under different temporal pressure conditions. Because negative events may occur unpredictably in our daily life, the prospective approach is beneficial for minimizing unfavorable consequences by predicting decision-making behaviors when actually encountering events based on psychological factors, such as anxious profiles in the present research. We assumed that pre-specified trait and state anxiety would differently predict decision-making performances, because trait anxiety may be sensitive to relatively remote, final decision-making outcomes concerning the overall task mission of maximizing final winnings, whereas state anxiety would likely be sensitive to decision-making immediately after negative events, specifically the maximum penalty events. To test the prediction, global and local behavioral indexes were calculated and regressed by the trait and state anxiety profiles assessed before the IGT was performed. Trait anxiety predicted global decision-making behaviors only in the self-paced condition without temporal pressure: higher trait anxiety predicted higher preference for overall high risk-taking in the self-paced condition. On the other hand, pre-specified state anxiety differently predicted local decision-making behaviors between the self- and forced-paced conditions: higher state anxiety predicted higher preference for risk-avoidance after the maximum penalty events in the self-paced condition, and on the other hand, predicted higher preference for moderate risk-taking in the forced-paced condition. These findings suggest that pre-specified trait and state anxiety work differently as predictors of future decision-making performance under different temporal pressure conditions.

Trait anxiety predicted global risk-taking behaviors in the self-paced condition: participants with higher trait anxiety more frequently selected the high-risk deck (Deck 2) and more frequently deselected the low-risk decks (Decks 3 and 4), thus encountering more maximum penalty events (-125,000 yen in Deck 2). These findings seemed to be counterintuitive at first glance, because trait anxiety tends to promote cognitive bias toward occurrences of negative events and risk-avoidance behaviors, as observed in people with elevated trait anxiety (Miu et al., 2008; Mueller et al., 2010). One of possible interpretation of the findings may be that participants with higher anxiety traits more frequently selected high-risk decks for recovering large losses even after facing to maximum penalty events. If they were provided with the instruction of the deck types and optimal strategies before the task, they might recognize that they should find out and continuously choose the advantage decks. Although participants might develop their own assumptions about the deck types even under information ambiguity as shown by self-reports of their deck-selection after the tasks, the procedure was likely different from confirmation or confidential decision-making (Vickers and Packer, 1982), in which participants are provided with prior information about the deck types and become confidentially convinced of it throughout the task. Poorer amounts of prior knowledge possibly induce higher anxiety with less confidence, as observed in, for example, verbal learning contexts (Yang and Quadir, 2018). Upon the present experimental context without the prior knowledge, participants might develop their own assumption about the deck types, which is indicated by the finding that the maximum penalties induced immediate risk-avoidance, as observed in Figure 5A,B. However, participants with, in particular, higher anxiety traits might be less confident of their strategic assumptions, and yield anxiety toward future, uncertain monetary gains, selecting high-reward decks for monetary recovery. This is suggested by the fact that the average net-scores in the self-paced condition did not gradually increase as represented by Figure 2, and actually, about 30% participants self-reported that they used the strategies to actively select high-reward decks to rapidly recover the largest loss or earn rewards as much as possible. It is speculated in summary that under information ambiguity, participants with higher trait anxiety were more anxious for future failure in maximizing final rewards and more frequently selected the high-reward decks even if they developed the assumption that the decks might sometime impose high penalties.

As revealed by comparing local deck-selections between the pre- and post-penalty events, post-penalty risk-avoidance was similarly promoted in both the self- and forced-paced conditions (Figure 5A,B). However, state anxiety predicted local decision-making performances differently in the self- and forced-paced conditions, which indicates that state anxiety profiles specified before the IGT are not irrelevant but continuous to future decision-making. In the self-paced condition, higher state anxiety predicted more frequent risk-avoidance after maximum penalty events: higher state anxiety was related to lower selectivity of the high-risk deck as well as higher selectivity of the low-risk decks, particularly after the second penalty event. As has been argued for global decision-making outcomes, the present participants, distinguished from individuals with abnormal emotional assessment and decision-making (Bechara et al., 2000a), could likely appropriately assess a somatic marker emotionally evoked by the maximum penalty events (Damasio, 1996) and regulate emotional response accordingly, thereby engaging in local risk-avoidance behaviors under no temporal pressure. Although decision-making in the IGT tends to be differentiated from general executive functions (Bechara et al., 1998; Bechara, 2004; Toplak et al., 2010), it requires suppressing emotional disturbance caused by the penalty events and monitoring deck selection by inhibiting the other options (Paulus, 2005), which may be related to dorsolateral prefrontal functions outside the ventromedial prefrontal areas (Ernst et al., 2002). Therefore, in the present study, the participants with higher state anxiety were sensitive to the maximum penalty events and appropriately drove executive functions without emotional disturbance, thereby transiently avoiding risk-taking more frequently. Considering the global behavioral findings together, the dynamic nature of decision-making without temporal pressure may be comprehensible as a function of the interaction between trait and state anxiety. Global decision-making behaviors were predicted by trait anxiety, which indicates that higher trait anxiety is related to higher risk-taking overall. However, local decision-making was sensitive to state anxiety and higher anxious states were related to higher risk-avoidance in an opposite manner. That is, participants with normal decision-making may have switched risk-taking and risk-avoidance adaptively under no temporal pressure according to changes in their anxiety profiles in a conflicting situation between the current penalty events and the remote task mission in the course of the IGT under information uncertainty without hints of deck types and an optimal strategy. Although the trait and state anxiety scores of the present participants yielded significant positive correlation (*r* = 0.458, *p* = 0.007), its strength was not prominently high, suggesting that trait and state anxiety possess multidimensionality and may not be strongly correlated because the current situation was not completely compatible with ordinary-life anxious conditions that the participants tend to face (Endler et al., 1991; Leal et al., 2017). Such mild correlation between the two anxiety profiles may in turn leave a margin for partial dissociation between them, consequently yielding a dynamism of global and local decision-making.

Under the forced-paced condition, state anxiety predicted intermediate risk-avoidance behaviors. That is, higher state anxiety was related to more frequent selection of the middle-risk deck. Similar to the self-paced condition, the forced-paced condition showed low selectivity of the high-risk deck and high selectivity of the low-risk decks as shown in Figure 5A,B. The participants, whether with high or low state anxiety, tended to locally avoid high risk-taking, but the participants with higher pre-specified state anxiety more frequently engaged in moderate risk-taking even after the maximum penalty under temporal pressure. There are two possible interpretations of the local decision-making pattern under temporal pressure based on different psychological backgrounds. The first interpretation concerns automatic emotional dysregulation. Decision-making in the IGT is related to two stages in the processing of somatic states (Bechara et al., 2003). In the first stage, the primary inducer is an external event and situation that automatically evokes a somatic state, such as the maximum penalty event in the present study. The secondary inducer includes psychological entities, such as thoughts and memories of emotional events and situations evoking primary somatic states. When facing the maximum penalty events under temporal pressure, the participants might have experienced somatic states through primary as well as secondary inducers in a complex manner and escaped from the current high risk-taking; however, they may not have completely regulated their emotional reactions, thereby automatically selecting the middle-risk deck as a consequence. The second interpretation is related to controlled compensation. Decision-making in the IGT generally comprises three stages: anticipatory option assessment, action execution, and outcome evaluation (Paulus, 2005). In particular, the latter two are related to the cognitive inhibition of competitive options and post-action monitoring as a general executive function (Fellows, 2007; Ouerchefani et al., 2017). The participants regulate emotional reactions to the maximum penalty events under temporal pressure to avoid selecting the high-risk deck; however, they might dare to actively select the middle-risk deck under attentional control to recover monetary loss as soon as possible. Because subsequent decision-making behaviors depend on monitoring response feedback (Yi et al., 2012), selecting the middle-risk deck under temporal pressure is possibly highly adaptive under behavioral monitoring for simultaneously coping with elevated anxiety states caused by the current penalty and sustained less-state anxiety concerning final winnings during the IGT, which would induce the negative consequences of larger monetary loss. At present, our findings cannot completely determine which mechanism is plausible for decision-making under the forced-paced condition because participants’ self-reports indicate that they voluntarily selected card decks based on control of their own developing strategy. On the other hand, unlike the self-paced condition, RTs under temporal pressure did not show a gradual reduction over the progress of trials likely based on an adaptive effect by learning the task (Visser et al., 2007). The suggestion is that salient penalty events implicitly promote cautious attitudes and does not fasten decision-making even in later trials under temporal pressure. The relation between conscious self-reports and RTs, which are not necessarily controlled-behavioral indexes, likely provides information about the adaptive aspects of decision-making in interactions between our internal and external states.

The implications of the present findings may be relevant to social problems such as billing frauds. In Japan, for example, the incidence of billing frauds has grown annually (the number of incidents in 2017 was 18,212, with an increase rate of about 30% compared to the previous year^1^), and the amount of monetary damage per incident was about 2,300,000 yen. Prevention measures for a billing fraud, therefore, are required not only at the public social level but also at the personal psychological level. The present findings for the IGT in the forced-paced condition may provide potential information for psychological prevention measures. The state anxiety of people who are vulnerable to future billing frauds may easily fluctuate under subjectively perceived temporal pressures that are externally evoked by defrauders, and these individuals may tend to falter in their emotional regulation, consequently transferring money to the defrauders’ bank accounts. Noticeably, the requested amounts of billing money are not too large to be paid (e.g., 2,300,000 yen in 2017 in Japan, a decrease compared to the previous year), that is, not high but moderate risk requirements. Therefore, we should focus on the psychological mechanisms of not only high risk-taking but also moderate risk-taking for exploring prevention measures.

Finally, the limitations of the present study should be discussed. State and trait anxiety were recorded on the same day as the IGT. Although trait anxiety is related to chronic anxiety properties observed in ordinary life, state anxiety is related to a current transient state of anxiety. Therefore, assessment of state anxiety might have occurred too soon before the IGT for prediction analysis of decision-making. A stricter methodology would have had participants undergo the IGT and STAI assessments multiple times on separate days. If the predictability of IGT performances was established via anxiety profiles collected on separated days, the reliability of the present results would be increased.

To conclude, the present study used the IGT to simulate several aspects of real-life decision-making processes and examined how anxiety profiles differently predicted future decision-making performances under different temporal pressure conditions. The prospective approach predicts decision-making performances based on the pre-specified psychological profiles of individuals and applying it may be beneficial for people to avoid socially and privately negative consequences. Pre-specified trait and state anxiety differently predicted future decision-making behaviors. The present study showed that under temporal pressure, moderate risk-taking rather than high risk-taking was enhanced after negative events by high sensitivity to state anxiety. The psychological mechanism for moderate risk-taking should be examined in future research, bearing in mind that “a small leak will sink a great ship.”

## Data Availability

All datasets generated for this study are included in the manuscript and/or the Supplementary Files.

## Ethics Statement

Written informed consent was obtained from the participants according to the institutional guidelines before conducting the experiment. The study was conducted in accordance with the Declaration of Helsinki, a statement of ethical principles for medical research involving human participants, and was approved by the Ethics Committee of the Tokyo Institute of Technology.

## Author Contributions

TS, MN, and AT designed the study. MN and EF performed data collection. TS and MN analyzed the data and wrote the initial version of the manuscript. TS, MN, EF, and AT revised the manuscript.

## Conflict of Interest Statement

The authors declare that the research was conducted in the absence of any commercial or financial relationships that could be construed as a potential conflict of interest.

## Footnotes

1 https://www.npa.go.jp/publications/statistics/sousa/sagi.html

## Supplementary Material

DATA SHEET S1 Data analyzed for participants’ global and local performances of the Iowa Gambling Task under the two different temporally pressured conditions.

FIGURE S1 Cumulative histogram of dummy *p*-values in the permutation regression analyses for the model with the independent variable STAI-T for the numbers of maximum penalty events (*n* = 100,000). Cumulative frequencies of dummy *p*-values gradually increase from zero to one in the horizonal *p*-value axis. The actual *p*-value of the significant model was *p* = 0.049 in the self-paced condition. The values was within the lower 5% of the distribution (*p* < 0.0506) and was certified as significance-corrected for multiple testing.

TABLE S1 Comparisons of global decision-making outcomes between the selfand forced-paced conditions (*n* = 33).

TABLE S2 Comparisons of selectivity (%) of the high-, middle-, and low-risk decks between pre- and post-penalty events in the self-paced condition.

TABLE S3 Comparisons of selectivity (%) of the high-, middle-, and low-risk decks between pre- and post-penalty events in the forced-paced condition.
