# Supplementary material for: Pre-specified Anxiety Predicts Future Decision-Making Performances Under Different Temporally Constrained Conditions
Source: Front Psychol. 2019 Jul 9;10:1544. doi: 10.3389/fpsyg.2019.01544 (PMC6634256; doi:10.3389/fpsyg.2019.01544)

Supplementary Table 1. Comparisons of global decision-making outcomes between the self- and forced-paced conditions (n = 33).

Performance index	Self-paced		Forced-paced		Wilcoxon test	
	Mean	*SE*	*Mean*	*SE*	*Z*-value	*p*-value
Overall trials						
Final winnings (Japanese yen)	−35,000	16,497	−38,485	22,507	0.617	0.537
Numbers of maximum penalty events	3.5	0.3	3.4	0.3	0.061	0.951
Mean entropy (bit)	1.82	0.18	1.79	0.26	0.524	0.600
High-risk deck						
Total selectivity (%)	34.3	2.7	30.7	2.5	0.804	0.422
Continuous selectivity (%)	16.2	2.3	15.8	2.7	0.253	0.801
Middle-risk deck						
Total selectivity (%)	19.8	1.1	19.5	1.5	0.175	0.861
Continuous selectivity (%)	26.8	3.0	27.0	3.8	0.170	0.865
Low-risk deck						
Total selectivity (%)	45.9	2.6	50.0	3.0	0.751	0.453
Continuous selectivity (%)	52.5	3.9	58.1	4.4	1.108	0.268

Supplementary Table 2. Comparisons of selectivity (%) of the high-, middle-, and low-risk decks between pre- and post-penalty events in the self-paced condition.

Trial phase	High-risk deck						Middle-risk deck						Low-risk deck					
	1st (n = 32)		2nd (n = 30)		3rd (n = 24)		1st (n = 32)		2nd (n = 30)		3rd (n = 24)		1st (n = 32)		2nd (n = 30)		3rd (n = 24)	
	Mean	*SE*	*Mean*	*SE*	*Mean*	*SE*	*Mean*	*SE*	*Mean*	*SE*	*Mean*	*SE*	*Mean*	*SE*	*Mean*	*SE*	*Mean*	*SE*
pre	43.8	6.0	37.3	5.0	47.5	5.9	22.5	3.4	22.0	3.6	18.3	3.2	33.8	5.1	39.4	3.8	31.5	5.1
post 1	25.6	5.0	22.0	4.5	31.7	5.6	24.4	4.1	28.7	3.9	24.2	5.0	50.0	4.7	47.7	4.5	40.8	5.9
post 2	30.0	3.7	39.3	5.5	36.7	6.0	17.5	2.8	13.3	2.4	18.3	4.2	52.5	4.1	42.6	5.5	37.7	5.8
Friedman test	χ^2-value	p-value	χ^2-value	p-value	χ^2-value	p-value	χ^2-value	p-value	χ^2-value	p-value	χ^2-value	p-value	χ^2-value	p-value	χ^2-value	p-value	χ^2-value	p-value
	3.11	0.211	7.22	0.027*	3.95	0.139	2.99	0.224	10.02	0.007**	1.11	0.573	9.05	0.011*	1.84	0.399	1.98	0.373
Wilcoxon test	Z-value	p-value	Z-value	p-value	Z-value	p-value	Z-value	p-value	Z-value	p-value	Z-value	p-value	Z-value	p-value	Z-value	p-value	Z-value	p-value
pre vs. post 1	-	-	2.331	0.020*	-	-	-	-	1.090	0.276	-	-	2.545	0.011*	-	-	-	-
pre vs. post 2	-	-	0.323	0.747	-	-	-	-	2.124	0.034	-	-	2 .911	0.004**	-	-	-	-

1st: the first maximum penalty event; 2nd: the second maximum penalty event; 3rd: the third maximum penalty event; pre: five trials before the penalty event; post 1: the first-half five trials after the penalty event; post 2: the second-half five trials after the penalty event; *$p < 0.05$; **$p < 0.01$

Supplementary Table 3. Comparisons of selectivity (%) of the high-, middle-, and low-risk decks between pre- and post-penalty events in the forced-paced condition.

Trial phase	High-risk deck						Middle-risk deck						Low-risk deck					
	1st (n = 30)		2nd (n = 30)		3rd (n = 24)		1st (n = 30)		2nd (n = 30)		3rd (n = 24)		1st (n = 30)		2nd (n = 30)		3rd (n = 24)	
	Mean	*SE*	*Mean*	*SE*	*Mean*	*SE*	*Mean*	*SE*	*Mean*	*SE*	*Mean*	*SE*	*Mean*	*SE*	*Mean*	*SE*	*Mean*	*SE*
pre	52.7	6.2	42.7	5.7	55.8	6.1	16.0	2.8	17.3	2.7	15.0	3.0	31.3	5.2	40.0	5.0	29.2	5.2
post 1	34.0	5.4	24.0	5.8	23.3	6.1	23.3	4.0	29.3	5.6	21.7	4.2	42.7	5.8	46.7	6.4	55.0	6.2
post 2	32.7	4.8	36.0	4.9	30.8	5.2	16.0	3.8	18.0	3.9	15.0	3.7	51.3	5.5	42.7	5.7	51.7	6.9
Friedman test	χ^2-value	p-value	χ^2-value	p-value	χ^2-value	p-value	χ^2-value	p-value	χ^2-value	p-value	χ^2-value	p-value	χ^2-value	p-value	χ^2-value	p-value	χ^2-value	p-value
	5.61	0.061	7.67	0.022*	11.61	0.003**	2.86	0.239	1.82	0.403	1.27	0.529	3.2	0.202	0.02	0.990	9.07	0.011*
Wilcoxon test	Z-value	p-value	Z-value	p-value	Z-value	p-value	Z-value	p-value	Z-value	p-value	Z-value	p-value	Z-value	p-value	Z-value	p-value	Z-value	p-value
pre vs. post 1	-	-	2.502	0.012*	3.090	0.002**	-	-	-	-	-	-	-	-	-	-	2.666	0.008**
pre vs. post 2	-	-	1.000	0.317	2.653	0.008**	-	-	-	-	-	-	-	-	-	-	2.388	0.017*

1st: the first maximum penalty event; 2nd: the second maximum penalty event; 3rd: the third maximum penalty event; pre: five trials before the penalty event; post 1: the first-half five trials after the penalty event; post2: the second-half five trials after the penalty event; *$p < 0.05$; **$p < 0.01$

Supplementary Figure 1. Cumulative histogram of dummy *p*-values in the permutation regression analyses for the model with the independent variable STAI-T for the numbers of maximum penalty events (n = 100,000). Cumulative frequencies of dummy *p*-values gradually increase from zero to one in the horizonal *p*-value axis. The actual *p*-value of the significant model was $p = 0.049$ in the self-paced condition. The values was within the lower 5% of the distribution ($p < 0.0506$) and was certified as significance-corrected for multiple testing.

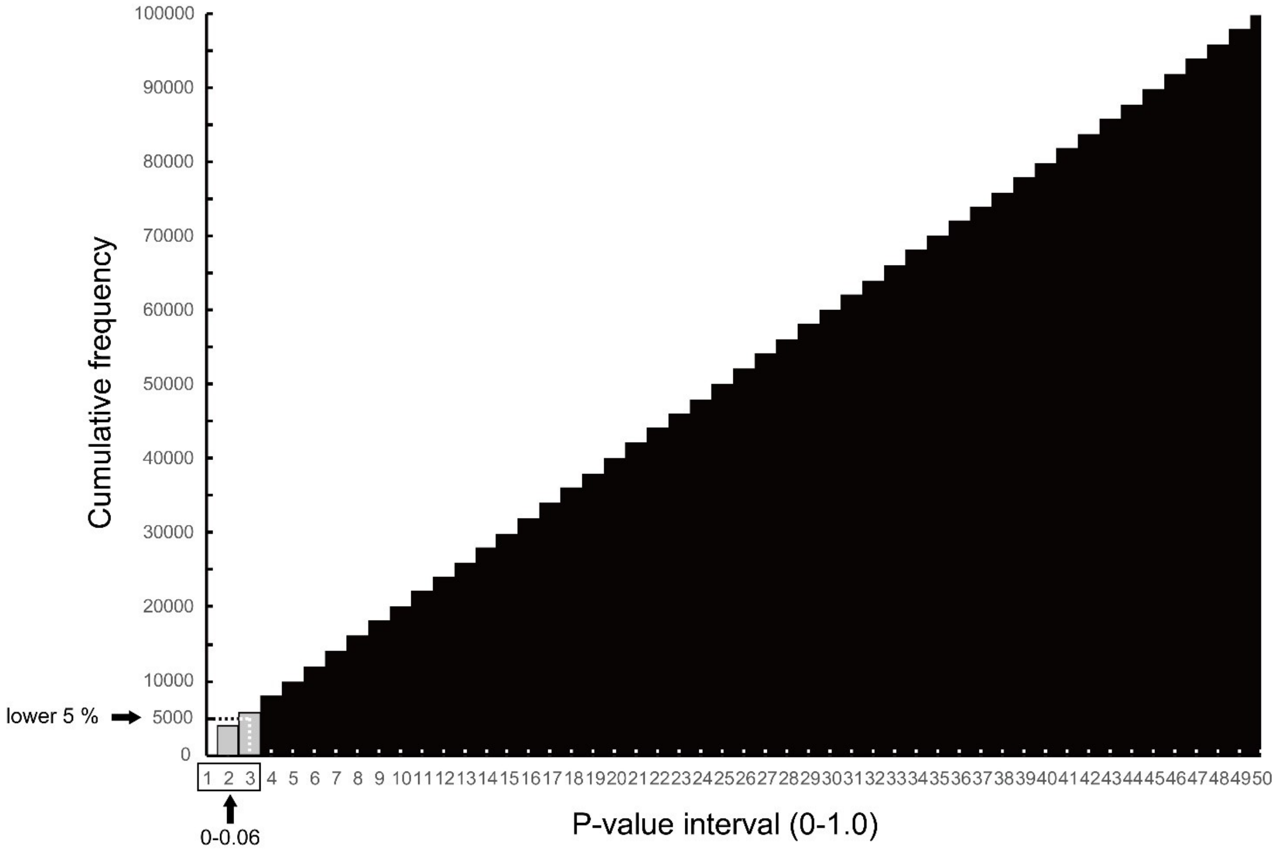

Supplement: FIGURE S1 — Cumulative histogram of dummy p-values in the permutation regression analyses for the model with the independent variable STAI-T for the numbers of maximum penalty events (n = 100,000). Cumulative frequencies of dummy p-values gradually increase from zero to one in the horizonal p-value axis. The actual p-value of the significant model was p = 0.049 in the self-paced condition. The values was within the lower 5% of the distribution (p < 0.0506) and was certified as significance-corrected for multiple testing. [file Data_Sheet_2.pdf]
